# Sociodemographic and economic characteristics of families and health and education conditions of children in the BRISA cohort during the COVID-19 pandemic

**DOI:** 10.1590/1980-549720230036

**Published:** 2023-08-28

**Authors:** Julia Hannah Teixeira, Paulo Ricardo Higassiaraguti Rocha, Eduardo Carvalho de Arruda Veiga, Karina Bezerra Salomão, Manuela Ramos Barbieri, Mariana Moraes de Oliveira, Viviane Cunha Cardoso, Ricardo de Carvalho Cavalli, Marco Antonio Barbieri, Maria da Conceição Pereira Saraiva, Heloisa Bettiol

**Affiliations:** I Universidade de São Paulo, Ribeirão Preto School of Nursing – Ribeirão Preto (SP), Brazil.; II Universidade de São Paulo, Faculty of Medicine of Ribeirão Preto – Ribeirão Preto (SP), Brazil.; III Universidade de São Paulo, Faculty of Dentistry of Ribeirão Preto – Ribeirão Preto (SP), Brazil.

**Keywords:** Cohort studies, Pandemics, Child health, Socioeconomic factors, Estudos de coortes, Pandemias, Saúde da criança, Fatores socioeconômicos

## Abstract

**Objective:**

To describe changes in sociodemographic, economic and variables related to the characterization of family, health and education during the COVID-19 pandemic in a birth cohort evaluated at 10–11 years of age.

**Methods:**

Cross-sectional study involving 1,033 children from a cohort of children born in 2010/2011, in the city of Ribeirão Preto, SP, Brazil. Data were collected from July to October 2021 by telephone or video interview held with the person responsible for the child. The questionnaires discussed family organization, child behavior and health, school attendance, socioeconomic assessment and occurrence of COVID-19 during the period of social isolation due to the pandemic. Descriptive statistics were used to describe the data. The chi-square test was used to verify group differences by minimum wages (MW).

**Results:**

Of the respondents, 47.6% reported worsening of their financial situation during the pandemic, which was more frequent in the group with a household income <3 MW compared to the group with >6 MW (59.1 *vs.* 15.7%; p<0.001). According to the respondents, 62% of the children exhibited behavioral changes during the period and anxiety was the most frequently reported condition. In addition, 61.4% of the children had learning difficulties and these problems were more prevalent among children from households with lower incomes compared to those with higher incomes (74.7 *vs.* 45.1%; p<0.001).

**Conclusion:**

The COVID-19 pandemic has changed different economic aspects of families, as well as educational, health and behavioral indicators of children. Lower-income families were the most affected both economically and in terms of other indicators.

## RESUMO

**Objetivo:**

Descrever as alterações nas características sociodemográficas, econômicas e variáveis relacionadas à caracterização da família, saúde e educação durante a pandemia da COVID-19, em uma coorte de nascimento avaliada aos 10–11 anos de idade.

**Métodos:**

Estudo transversal envolvendo 1.033 crianças de uma coorte de nascidos em 2010/2011, na cidade de Ribeirão Preto, SP, Brasil. Os dados foram obtidos por meio de entrevistas por telefone ou videochamada com o responsável pela criança, no período de julho a outubro de 2021. Os questionários abordaram informações sobre a organização familiar, comportamento e saúde da criança, acompanhamento escolar, avaliação socioeconômica e ocorrência da COVID-19 durante o período de isolamento social. Foi realizada estatística descritiva. O teste qui-quadrado foi utilizado para verificar diferenças de grupos por salários-mínimos (SM).

**Resultados:**

Dos entrevistados, 47,6% relataram piora na condição financeira durante a pandemia, sendo a piora econômica mais frequente no grupo de renda familiar <3 SM em comparação ao grupo de >6 SM (59,1 *vs.* 15,7%; p<0,001). Segundo os responsáveis, 62% das crianças apresentaram mudança de comportamento durante o período, sendo a ansiedade relatada com mais frequência. Ainda, 61,4% das crianças apresentaram dificuldades de aprendizagem, e o prejuízo foi mais acentuado naquelas de menor renda familiar em comparação às de maior renda (74,7 *vs.* 45,1%; p<0,001).

**Conclusão:**

A pandemia da COVID-19 alterou diferentes aspectos econômicos das famílias, assim como indicadores educacionais, de saúde e de comportamento das crianças. As famílias de menor renda foram as mais prejudicadas tanto do ponto de vista econômico como nos demais indicadores.

## INTRODUCTION

The end of 2019 was marked by a pneumonia outbreak of unknown origin in the province of Wuhan, China. Shortly thereafter, in January 2020, the cause of the outbreak was discovered and was characterized as a new member of the coronavirus family, SARS-CoV-2. The disease was called COVID-19. On March 11, 2020, the World Health Organization declared COVID-19 a new pandemic, which has had major health impacts and changed global social and economic scenarios^1,2^.

The measures taken to contain the pandemic in Brazil included the implementation of social distancing and, in positive cases, compliance with quarantine rules, which had an impact on the country’s economic activities^3^. The effects of the economic scenario affected the population’s way of life, with implications for health and well-being, and revealed still existing inequalities^4^.

COVID-19 affects all age groups, including children whose symptoms vary depending on the region where this population lives and on the virus variant^5,6^. The pandemic has demonstrated even more latent and habitual issues that involve socioeconomic, political, cultural and ethnic inequalities, accentuating challenges in the field of public health^7,8^. The impacts of the disease go beyond clinical conditions and reach social and psychological issues, permeating social vulnerabilities in Brazil^9^.

Several studies have demonstrated the impact of the pandemic on the physical and mental health of children^10–13^. Within the educational context, remote teaching was adopted in order to avoid the agglomeration of children inside the school, which led to methodological changes in teaching-learning and unstructured social interactions^14–16^. There were impacts on the income of Brazilian families, affecting the household’s sources of income and causing financial and emotional instabilities, in addition to health-related losses^4^.

In view of the evident changes that have occurred in the world and in Brazil as a result of the COVID-19 pandemic, studies investigating the impacts of this period on sociodemographic, economic, behavioral and health conditions in the population, especially children, are important^17,18^. Therefore, the present study describes the changes in sociodemographic and economic variables, as well as those related to the characterization of family, health and education that occurred during the COVID-19 pandemic in a Brazilian birth cohort started in 2010, considering that at this stage of life (10–11 years) children are in an important developmental window of opportunity. In addition, the data will make it possible to investigate the impact of the pandemic on future outcomes, since the population of this research is part of a cohort that will be followed up at other times throughout life.

## METHODS

### BRISA cohort

In 2010, the project “Etiological factors of preterm birth and consequences of perinatal factors in child health: birth cohorts in two Brazilian cities — BRISA” (acronym for Brazilian Ribeirão Preto and São Luís Birth Cohort Studies) was started in two Brazilian municipalities with contrasting socioeconomic and demographic characteristics: Ribeirão Preto, state of São Paulo, and São Luís, state of Maranhão. The BRISA project comprised two cohorts in each city: a prenatal cohort^19^ (convenience sample of 1,400 pregnant women in Ribeirão Preto) started during pregnancy, and a birth cohort (population sample including 7,752 live births in Ribeirão Preto in 2010, corresponding to 95.7% of all births during this period in the municipality)^20^ that was followed up after the child’s birth. The main objective of the BRISA project was to investigate new risk factors for preterm birth, perinatal health indicators, and the impact of preterm birth and other factors on the growth and development of children. The present study only uses data from the Ribeirão Preto cohort. The first follow-up of children of the two cohorts occurred from 2011 to 2013 at 13 to 38 months of age^20^.

The second follow-up of the Ribeirão Preto BRISA cohort at school age (10–11 years) was scheduled to start in April/May 2020. However, because of restrictions due to the COVID-19 pandemic, by March 2020, face-to-face assessments had been postponed until a vaccine was available for a good portion of the population and greater pandemic control. Nevertheless, we believed that assessment during social isolation would be important for understanding the short- and long-term impacts of the pandemic on children and their families. Within this context, data from a cohort study would permit to evaluate the effect of the pandemic on different outcomes over time. Therefore, in order to investigate the effect of social restriction on socioeconomic indicators and family-, health- and education-related variables, in March 2021 we started to plan the BRISA Web study in this municipality, in which interviews were held remotely with the responsible persons.

### BRISA web

This was a cross-sectional study, in which all guardians or primary caregivers of children in the BRISA project were eligible for the present study. Based on the contact information from the previous phases of the project, the responsible persons were first contacted by telephone or via social networks in order to remind them about the BRISA project and to schedule an interview by video call or phone regarding issues related to the impact of the pandemic on socioeconomic indicators and variables related to the child’s family, health and education. For this purpose, a researcher of the team previously trained in the application of the questionnaire contacted the responsible person or primary caregiver of the child on the scheduled day and time to start the interview. Each interview lasted approximately 25 minutes and the participant could ask to interrupt it at any time. The data were collected between 22 July and 19 October 2021 and were entered online into the REDCap electronic data capture tools^21,22^ during the interview. This online data collection was conducted at the Clinical Research Unit (UPC) of the University Hospital, Ribeirão Preto Medical School, University of São Paulo (HCFMRP-USP).

The procedures of this phase of the BRISA project were approved by the Ethics Committee of HCFMRP-USP (Approval number 4.826.298).

### Variables

The questionnaire was applied in the form of blocks that focused on family organization, child behavior and health, school attendance, nutrition, socioeconomic assessment, occurrence of COVID-19, sleep quality, and oral health (Supplementary Material).

The following information was collected to assess the sociodemographic and economic characteristics of the families: total household income in minimum wages (MW), in this case R$ 1,100.00 (<3, 3 to 6, >6); financial situation during the pandemic (improved, stayed the same, worsened); receiving donations from relatives (yes, no); lack of food and money to buy more food (yes, no); loss of job since the beginning of the pandemic (yes, no, no/ self-employed), and cancellation or change of health insurance to a cheaper provider (yes, no, did not have health insurance).

The following questions were applied to evaluate behavioral and educational indicators of the children: Was there any change in the child’s behavior during the pandemic? (yes, no); Did the child get more agitated during the pandemic? (yes, no); Did the child get more anxious during the pandemic? (yes, no); Did the child become more depressed during the pandemic? (yes, no); Did the child get more nervous during the pandemic? (yes, no); Did these difficulties affect the child’s daily life? (yes, no); What were the reasons for the changes in the child’s behavior during the pandemic? (staying at home for a long time, fear of the disease, lack of contact with other children, other); Was there any change in the child’s eating behavior? (yes, no); Was there any change in the amount of food consumed? (eating the same amount, eating more, eating less); Did the child change the consumption of foods with added sugar during the pandemic? (did not consume/reduced, stayed the same, increased); Did the child have learning difficulties during the pandemic? (yes, no); How was screen time use compared to the period before the pandemic (a lot more, slightly more, the same as before the pandemic, less, no flat screen equipment).

The following questions were applied regarding health and the occurrence of COVID-19: Did the child fail to take any vaccine of the vaccination scheme? (yes, no); Did the child fail to attend a routine medical appointment? (yes, no); Was there any change in the child’s weight? (no, gained weight, lost weight); How was the quality of sleep? (better, stayed the same, worse); Did the child fail to attend routine appointments or interrupted any dental treatment? (yes, no, no follow-up); Did the child have COVID-19? (yes, no); Did the child have symptoms? (yes, no); Did anyone living with the child have COVID-19? (yes, no); Was anyone living with the child hospitalized due to COVID-19? (yes, no); In addition to the people who live with the child, was a close relative hospitalized or did a close relative die from COVID-19? (yes, no).

### Data analysis

Descriptive statistics were used to describe the data, with the calculation of absolute and relative frequencies and 95% confidence intervals. Comparison of proportions between covariates by minimum wage range (<3, 3 to 6, >6) was performed by the chi-squared test and the level of significance was set at <0.05. The exposure MW and three main outcomes (change in the family’s financial situation, learning difficulties and weight gain of the child during social isolation) were considered for the sample calculation. Assuming that approximately 40% of the population would have income < 3 MW (Group 1), 30% between 3 to 6 MW (Group 2) and 10% above 6 MW (Group 3), with the expectation of achieving differences in outcomes of at least 10% among groups with test power of 80% and alpha of 0.05, the highest estimate among the three groups was 388 people per group comparing group 1 *versus* the others, and 89 comparing group 1 *versus* group 2, and 18 comparing group 1 *versus* group 3. Data were exported from REDCap to an electronic spreadsheet and analyzed using the Stata 14 program (College Station, Texas, USA).

## RESULTS

**Figure 1:**
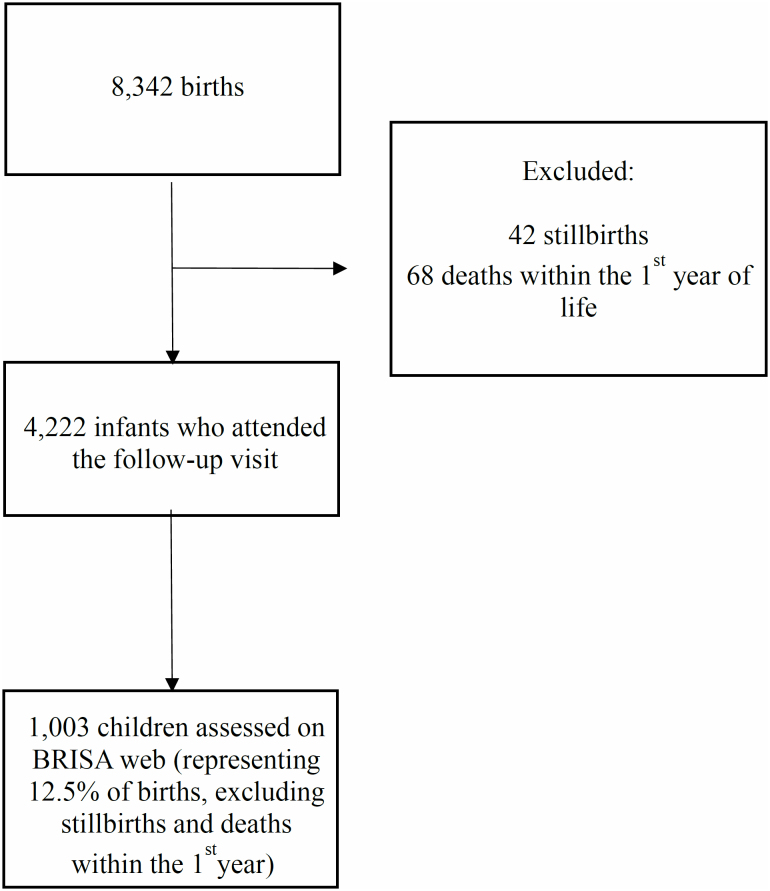
Flowchart of participant selection from the BRISA cohort database (Ribeirão Preto, SP, Brazil, 2010/2011).

The BRISA Web project evaluated 1,033, representing 12.5% of the total assessed at birth in both cohorts (prenatal cohort and birth cohort) (Figure 1). There were 15 twins (six twin pairs and one triplet). Only three of the 1,033 participants did not complete the questionnaire.

Table 1 shows the sociodemographic and economic characteristics of the families. Among the participants, 49% had a household income <3 minimum wages and 47.6% reported worsening of the financial situation during the pandemic. More than 1/4 (27.8%) of the respondents lost their job during the period and 11.5% reported at least one episode of not having money to buy food. In addition, 12.6% of the respondents had to cancel or change their health insurance to a cheaper provider during the pandemic.

**Table 1:** Absolute and relative frequency of sociodemographic and economic characteristics of the families.

Question		n	%	95%CI
Child sex				
	Female	494	47.8	44.8–50.9
	Male	539	52.2	49.1–55.2
Total household income (minimum wages)				
	>6	172	18.8	16.4–21.4
	3 to 6	295	32.2	29.3–35.3
	<3	449	49.0	45.8–52.3
Did your financial situation change during the pandemic?				
	Improved	95	9.3	7.6–11.2
	Stayed the same	441	43.1	40.1–46.2
	Worsened	486	47.6	44.5–50.6
Did the family receive donations (e.g., basic food basket, gas, clothes)?				
	Yes	238	23.3	20.8–26.0
	No	784	76.7	74.0–79.2
Did you ever run out of food and did not have the money to buy more?				
	Yes	118	11.5	9.7–13.6
	No	904	88.5	86.3–90.3
Has anyone lost a job since the beginning of the pandemic?				
	Yes	284	27.8	25.1–30.6
	No	705	69.0	66.1–71.7
	No, self-employed	33	3.2	2.3–4.5
Did you have health insurance that you had to cancel or change to a cheaper provider?				
	Yes	129	12.6	10.7–14.8
	No	507	49.6	46.5-52.7
Did not have health insurance		386	37.8	34.8–40.8

Differences in the total number in relation to the reference (n) are due to missing information. 95%CI: 95% confidence interval.

Approximately 2/3 (62.1%) of the respondents reported changes in the child’s behavior during the pandemic (Table 2). Anxiety (92.7%), nervousness (62.6%) and agitation (59.8%) were the most frequent changes during isolation and the main causes were staying at home for a long time (44.9%) and not having contact with other children (31.1%). Regarding dietary habits, 2/3 (65.5%) of the respondents identified changes in the child’s eating behavior, with more than half of the children (52.1%) eating more and 39.0% consuming more sweets. Most children (61.4%) had learning difficulties during the pandemic. The majority of respondents (92.5%) revealed that children spent more time in front of screens compared to the period before the pandemic.

**Table 2:** Absolute and relative frequency of behavioral and health variables of children during the pandemic.

Questions		n	%	95%CI
Was there any change in the child’s behavior during the pandemic?				
	Yes	641	62.1	59.0–65.0
	No	392	37.9	35.0–41.0
Did the child get more agitated during the pandemic?				
	Yes	383	59.8	55.9–63.5
	No	258	40.3	36.5–44.1
Did the child get more anxious during the pandemic?				
	Yes	594	92.7	90.4–94.5
	No	47	7.3	5.5–9.6
Did the child become more depressed during the pandemic?				
	Yes	242	37.7	34.1–41.6
	No	399	62.3	58.4–65.9
Did the child get more nervous during the pandemic?				
	Yes	401	62.6	58.7–66.2
	No	240	37.4	33.8–41.3
Did these difficulties affect the child’s daily life?				
	Yes	428	67.0	63.2–70.5
	No	211	33.0	29.5–36.8
What was the reason for the changes in the child’s behavior during the pandemic?				
	Staying at home for a long time	287	44.9	41.1–48.8
	Fear of the disease	35	5.5	39.5–75.4
	Not having contact with other children	199	31.1	27.7–34.8
	Other	118	18.5	15.6–21.7
Was there any change in the child’s eating behavior?				
	Yes	675	65.5	62.6–68.4
	No	355	34.5	31.6–37.4
Regarding the amount of food, the child is…				
	Eating the same amount	444	43.1	40.1–46.2
	Eating more	536	52.1	49.0–55.1
	Eating less	49	4.8	3.6–6.2
Did the child change the consumption of food with added sugar during the pandemic?				
	Did not consume/reduced	62	6.0	4.7–7.6
	Stayed the same	566	55.0	51.9–58.0
	Increased	402	39.0	36.1–42.1
Did the child have learning difficulties during the pandemic?				
	Yes	631	61.4	58.4–64.4
	No	396	38.6	35.6–41.6
How was screen time use compared to the period before the pandemic?				
	A lot more	796	77.3	74.6–79.7
	Slightly more	157	15.2	13.2–17.6
	Stayed the same as before the pandemic	64	6.2	4.9–7.9
	Less	11	1.1	0.6–1.9
	No flat screen equipment	2	0.2	0.04–0.8
Did the child fail to take any of the scheduled vaccines?				
	Yes	131	12.7	10.7–14.8
	No	902	87.3	85.1–89.2
Did the child fail to attend any routine medical appointment?				
	Yes	460	44.5	41.5–47.6
	No	573	55.5	52.4–58.5
Did you notice any change in the child’s weight?				
	No	455	44.3	41.2–47.3
	Yes, gained weight	508	49.4	46.4–52.5
	Yes, lost weight	65	6.3	5.0–8.0

Differences in the total number in relation to the reference (n) are due to missing information. 95%CI: 95% confidence interval.

Regarding health-related variables, 12.7% of the children failed to take some vaccine of the vaccination scheme because of the pandemic and an important percentage (44.5%) did not attend routine medical appointments during the period. In addition, 49.4% of the respondents reported weight gain of the child during social isolation (Table 2).

**Table 3:** Absolute and relative frequency of COVID-19-related variables.

Questions		n	%	95% CI
Did the child have COVID-19?				
	Yes	78	7.7	6.2–9.5
	No	928	92.3	90.4–93.7
Did the child have symptoms?				
	Yes	64	82.1	71.7–89.2
	No	14	17.9	10.8–28.3
Did anyone living with the child have COVID-19?				
	Yes	405	39.7	36.7–42.7
	No	615	60.3	57.2–63.2
Was anyone living with the child hospitalized due to COVID-19?				
	Yes	42	10.4	7.7–13.7
	No	363	89.6	86.2–92.2
In addition to the people who live with the child, was a close relative hospitalized or did one die from COVID-19?				
	Yes	212	20.7	18.3–23.3
	No	812	79.3	76.7–81.7

Differences in the total number in relation to the reference (n) are due to missing information. 95% CI: 95% confidence interval.

Among the children, 7.7% had COVID-19 and 82.1% of them developed symptoms. Among household members, 39.7% had COVID-19 and 10.4% were hospitalized. Furthermore, 20.7% of the respondents reported hospitalization or death of a close relative due to COVID-19 (Table 3).

Learning difficulties (74.7%), non-attendance of medical (53.1%) or dental (32.9%) appointments, weight gain (53.1%), and worsening of sleep quality (39.8%) were more frequent among children from households with an income <3 minimum wages when compared to children from higher-income households (Table 4).

**Table 4:** Behavioral and health characteristics of the children according to household income in minimum wages.

Questions		Minimum wage							
		<3		3 to 6		>6		p-value*
		n	%	n	%	n	%	
Did your financial situation change during the pandemic?								
	Improved	31	6.9	125	42.4	40	23.3	<0.001
	Stayed the same	149	33.2	144	48.8	105	61.0	
	Worsened	269	59.1	26	8.8	27	15.7	
Has anyone lost a job since the beginning of the pandemic?								
	Yes	163	36.3	71	24.1	19	11.0	<0.001
	No	270	60.1	215	72.9	151	87.8	
	No, self-employed	16	3.6	9	3.0	2	1.2	
Did the child have learning difficulties during the pandemic?								
	Yes	334	74.7	158	53.4	79	45.1	<0.001
	No	113	25.3	138	46.6	96	54.9	
Did the child fail to attend any routine medical appointment?								
	Yes	239	53.1	121	40.7	53	30.3	<0.001
	No	211	46.9	176	59.3	122	69.7	
Did you notice any change in the child’s weight?								
	No	178	39.7	127	42.9	93	53.1	0.034
	Yes, gained weight	238	53.1	150	50.7	76	43.4	
	Yes, lost weight	32	7.1	19	6.4	6	3.4	
How is the sleep quality during this pandemic year?								
	Better	53	11.8	30	10.1	12	6.9	<0.001
	Stayed the same	218	48.4	176	59.3	121	69.1	
	Worse	179	39.8	91	30.6	42	24.0	
Did the child fail to attend appointments or interrupt any dental treatment?								
	Yes	148	32.9	81	27.3	47	26.9	<0.001
	No	239	53.1	202	68.0	126	72.0	
	No dental follow-up	63	14	14	4.7	2	1.1	

Differences in the total number in relation to the reference (n) are due to missing information.

* chi-square test.

## DISCUSSION

The data of the present study reveal that the COVID-19 pandemic changed different economic aspects of families, as well as indicators of children’s health and behavior. For 47.6% of respondents, there was a worsening in the family’s financial situation during the period of social isolation. Yet, the fact that the child remained indoors for long periods resulted in important behavioral changes, often observed in this period, such as increased anxiety and nervousness, which hindered the performance of day-to-day activities. In general, it could be seen that families classified in the lowest income range (<3 MW) were the most affected during social isolation.

The COVID-19 pandemic has substantially affected the financial situation of families, with approximately half of the respondents reporting worsening of their economic situation during the period of isolation. This worsening was even more pronounced among participants with lower household incomes, who lost their jobs more frequently during the period compared to the higher-income group. The consequences are reflected in the other variables analyzed, such as lack of money to buy food and a decline in food variety. These data reinforce that economic difficulties were more intense among low-income families, a fact that may have further aggravated socioeconomic inequalities^4^.

Different studies have shown an increase in the prevalence of cases of anxiety and depression among children and adolescents during isolation^10,23,24^. This was also observed in the present study, in which anxiety was the most frequent behavioral change, followed by nervousness and depressive behaviors. Despite the well-known resilience of children and adolescents exposed to traumatic events such as social isolation, monitoring the mental health of this group is essential since the alleviation of symptoms developed during this period may take some years^25,26^. Furthermore, in the present study, approximately one-third of the families had a household member or close relative who was hospitalized and/or died from COVID-19, highlighting the importance of social assistance and support for children and their families.

The period of social isolation significantly altered the teaching-learning methods for children and adolescents^27^, causing learning difficulties, as observed in the present study, in about 2/3 of the children. These learning difficulties were even greater for children in the lower-income group compared to the other income groups. Some factors may explain this disparity between groups: difficulty in internet access, lack of quality electronic equipment, and lack of an appropriate place to watch the classes. In addition, Brazil was one of the countries where remote or hybrid teaching (remote and face-to-face) persisted for the longest period of time, with private schools returning to face-to-face teaching before public schools. Some studies have demonstrated the negative impact of remote teaching on learning and school dropout^15,16^. Lichand et al.^15^ observed a delay of approximately 75% in learning and an estimated school dropout rate of 35% in the state of São Paulo during the pandemic. In the present study, 80.6% of the children in the lowest income group (<3 minimum wages) attended public schools.

According to the respondents, almost half the children failed to attend their routine medical appointment. This fact implies a lack of follow-up and guidance to improve health indicators and may have contributed to the non-updating of the vaccination scheme in approximately 13% of the population and to the weight gain of children above the expected, as reported by approximately 50% of the responsible persons. In general, damage to health indicators such as a change in the child’s weight, worsening of sleep quality and non-attendance of medical and dental appointments was greater in children with a household income <3 minimum wages. These findings reinforce the need to target public health policies more intensely in the most economically vulnerable groups over the coming years^8^.

Approximately 8% of the children had COVID-19, with the majority of the cases developing symptoms. In a population-based study, Musa et al.^28^ found that only 40% of children aged 10 to 18 years diagnosed with COVID-19 were symptomatic, while 60% were asymptomatic. Thus, the percentage of children who had asymptomatic COVID-19 infection in the present study (17.9%) was probably underestimated.

The present study has some limitations in terms of the data collection process. The reports were obtained one year after the outbreak of the pandemic, in 2020, and the information was self-reported by the participants, which may have caused memory and information bias. Nevertheless, the data were collected by experienced and trained researchers through interviews conducted by telephone or video, thus minimizing possible failure of the respondents to understand certain questions and reducing cases of missing data, which may occur in the case of self-administered questionnaires. A strength of the study worth noting is that the population studied was a population-based birth cohort that will continue to be followed up over time; the data will therefore permit to investigate the impact of the pandemic on future outcomes.

In conclusion, the COVID-19 pandemic generally caused economic impacts on families, with those whose income was <3 minimum wages being the most affected. Likewise, damage to behavioral, educational and health indicators was more pronounced in children from lower-income households. In summary, education- and health-related public policies that address and consider the social inequalities, vulnerabilities and inequities intensified during the COVID-19 pandemic are needed.

## References

[1] World Health Organization, Emergency Committee (2020). Statement on the second meeting of the International Health Regulations
(2005) Emergency Committee regarding the outbreak of novel coronavirus
(2019-nCoV).

[2] World Health Organization (2020). WHO Director – General’s opening remarks at the media briefing on
COVID-19 – 11 March 2020.

[3] Souza JRC, Cavalcanti MAFH, Levy PM (2020). Visão geral da conjuntura. Instituto de Pesquisa Econômica
Aplicada [Internet]. Carta de Conjuntura.

[4] Almeida WS, Szwarcwald CL, Malta DC, Barros MBA, Souza PRB, Azevedo LO (2020). Mudanças nas condições socioeconômicas e de saúde dos brasileiros
durante a pandemia de COVID-19. Rev Bras Epidemiol.

[5] Instituto Butantan (2022). Crianças desenvolvem sintomas diferentes da ômicron; irritação na pele
está entre eles [Internet].

[6] Zare-Zardini H, Soltaninejad H, Ferdosian F, Hamidieh AA, Memarpoor-Yazdi M (2020). Coronavirus disease 2019 (COVID-19) in children: prevalence,
diagnosis, clinical symptoms, and treatment. Int J Gen Med.

[7] Matta GC, Rego S, Souto EP, Segata J, Matta GC, Rego S, Souto EP, Segata J (2021). Os impactos sociais da Covid-19 no Brasil: populações vulnerabilizadas e
respostas à pandemia.

[8] Ribeiro KB, Ribeiro AF, Veras MASM, Castro MC (2021). Social inequalities and COVID-19 mortality in the city of São
Paulo, Brazil. Int J Epidemiol.

[9] Morais AC, Miranda JOF (2021). Repercussões da pandemia na saúde das crianças brasileiras para
além da Covid-19. Physis (Rio J.).

[10] Jiao WY, Wang LN, Liu J, Fang SF, Jiao FY, Pettoello-Mantovani M (2020). Behavioral and emotional disorders in children during the
COVID-19 epidemic. J Pediatr.

[11] Lin JE, Asfour A, Sewell TB, Hooe B, Pryce P, Earley C (2021). Neurological issues in children with COVID-19. Neurosci Lett.

[12] Aydogdu ALF (2020). Saúde mental das crianças durante a pandemia causada pelo novo
coronavírus: revisão integrativa. J Health NPEPS.

[13] Barros MBA, Lima MG, Malta DC, Azevedo RCS, Fehlberg BK, Souza PRB (2022). Mental health of Brazilian adolescents during the COVID-19
pandemic. Psychiatry Res Commun.

[14] Andrew A, Cattan S, Dias MC, Farquharson C, Kraftman L, Krutikova S (2020). Inequalities in children’s experiences of home learning during
the COVID-19 lockdown in England. Fisc Stud.

[15] Lichand G, Doria CA, Leal O, Fernandes JPC (2022). The impacts of remote learning in secondary education during the
pandemic in Brazil. Nat Hum Behav.

[16] Moscoviz L, Evans DK (2022). Learning loss and student dropouts during the COVID-19 pandemic:
a review of the evidence two years after schools shut down
[Internet]. Working Paper 609.

[17] Wang G, Zhang Y, Zhao J, Zhang J, Jiang F (2020). Mitigate the effects of home confinement on children during the
COVID-19 outbreak. Lancet.

[18] Araújo LA, Veloso CF, Souza MC, Azevedo JMC, Tarro G (2021). The potential impact of the COVID-19 pandemic on child growth and
development: a systematic review. J Pediatr (Rio J).

[19] Silva AAM, Simões VMF, Barbieri MA, Cardoso VC, Alves CMC, Thomaz EBAF (2014). A protocol to identify non-classical risk factors for preterm
births: the Brazilian Ribeirão Preto and São Luís prenatal cohort
(BRISA). Reprod Health.

[20] Confortin SC, Ribeiro MRC, Barros AJD, Menezes AMB, Horta BL, Victora CG (2021). RPS Brazilian Birth Cohort Consortium (Ribeirão Preto, Pelotas
and São Luís): history, objectives and methods. Cad Saúde Pública.

[21] Harris PA, Taylor R, Thielke R, Payne J, Gonzalez N, Conde JG (2009). Research electronic data capture (REDCap)--a metadata-driven
methodology and workflow process for providing translational research
informatics support. J Biomed Inform.

[22] Harris PA, Taylor R, Minor BL, Elliott V, Fernandez M, O’Neal L (2019). The REDCap consortium: building an international community of
software partners. J Biomed Inform.

[23] Almeida ILL, Rego JF, Teixeira ACG, Moreira MR (2021). Social isolation and its impact on child and adolescent
development: a systematic review. Rev Paul Pediatr.

[24] Paiva ED, Silva LR, Machado MED, Aguiar RCB, Garcia KRS, Acioly PGM (2021). Child behavior during the social distancing in the COVID-19
pandemic. Rev Bras Enferm.

[25] Brooks SK, Webster RK, Smith LE, Woodland L, Wessely S, Greenberg N (2020). The psychological impact of quarantine and how to reduce it:
rapid review of the evidence. Lancet.

[26] Rider EA, Ansari E, Varrin PH, Sparrow J (2021). Mental health and wellbeing of children and adolescents during
the covid-19 pandemic. BMJ.

[27] Brasil. Ministério da Educação (2020). Gabinete do Ministro. Portaria no 544, de 16 de junho de 2020.
Dispõe sobre a substituição das aulas presenciais por aulas em meios
digitais, enquanto durar a situação de pandemia do novo coronavírus -
Covid-19, e revoga as Portarias MEC no 343, de 17 de março de 2020, no 345,
de 19 de março de 2020, e no 473 de 12 de maio de 2020
[Internet]. Diário oficial da União.

[28] Musa OAH, Chivese T, Bansal D, Abdulmajeed J, Ameen O, Islam N (2021). Prevalence and determinants of symptomatic COVID-19 infection
among children and adolescents in Qatar: a cross-sectional analysis of 11
445 individuals. Epidemiol Infect.

